# Molecular targets of spermidine: implications for cancer suppression

**DOI:** 10.15698/cst2023.07.281

**Published:** 2023-07-10

**Authors:** Andreas Zimmermann, Sebastian J. Hofer, Frank Madeo

**Affiliations:** 1 Institute of Molecular Biosciences, NAWI Graz, University of Graz, Graz, Austria.; 2 Field of Excellence BioHealth, University of Graz, Graz, Austria.; 3 BioTechMed Graz, Graz, Austria.

**Keywords:** spermidine, cancer, molecular targets, immunosurveillance, hypusination, autophagy

## Abstract

Spermidine is a ubiquitous, natural polyamine with geroprotective features. Supplementation of spermidine extends the lifespan of yeast, worms, flies, and mice, and dietary spermidine intake correlates with reduced human mortality. However, the crucial role of polyamines in cell proliferation has also implicated polyamine metabolism in neoplastic diseases, such as cancer. While depleting intracellular polyamine biosynthesis halts tumor growth in mouse models, lifelong external spermidine administration in mice does not increase cancer incidence. In contrast, a series of recent findings points to anti-neoplastic properties of spermidine administration in the context of immunotherapy. Various molecular mechanisms for the anti-aging and anti-cancer properties have been proposed, including the promotion of autophagy, enhanced translational control, and augmented mitochondrial function. For instance, spermidine allosterically activates mitochondrial trifunctional protein (MTP), a bipartite protein complex that mediates three of the four steps of mitochondrial fatty acid (β-oxidation. Through this action, spermidine supplementation is able to restore MTP-mediated mitochondrial respiratory capacity in naïve CD8^+^ T cells to juvenile levels and thereby improves T cell activation in aged mice. Here, we put this finding into the context of the previously described molecular target space of spermidine.

## INTRODUCTION

Polyamines are polycationic small molecules produced endogenously via the decarboxylation of the non-proteinogenic amino acid ornithine by ornithine decarboxylase (ODC). The product of this reaction, putrescine, is converted to spermidine and spermine by the stepwise addition of propylamine groups via spermidine (SRM) and spermine synthases (SMS), respectively. In addition, cellular polyamine pools are regulated by dietary uptake, microbial production and degradation [1]. Polyamine levels are homeostatically controlled by tight regulatory networks that quickly fine-tune endogenous production to the cellular supply.

Despite this stringent regulation, multiple reports, including a recent study by Al-Habsi *et al.* [2], show that polyamine levels, especially spermidine, decline significantly in different cell types and tissues of aging organisms [1]. While the molecular etiology of this phenomenon remains elusive, spermidine supplementation has emerged as a promising strategy to revert aging-associated cellular and organismal dysfunction [3–5]. Spermidine promotes several cellular processes that are linked to longevity, e.g., (i) autophagy, (ii) protein synthesis efficiency, and (iii) mitochondrial function. The latter results in increased oxidative phosphorylation rates and improved adenosine triphosphate (ATP) production. Although these processes are intertwined through several mechanistic nodes (e.g., mitochondrial oxidative phosphorylation depends on autophagy as well as spermidine-mediated translation efficiency) [4, 6], a universal molecular target that integrates the heterogenous cellular effects of spermidine has not been identified and might well be cell type- and tissue-specific. We have recently summarized the complexity of molecular interactors targeted by spermidine in the context of aging and autophagy [7]. While the exact hierarchical clustering of specific and relevant molecular factors for the lifespan-extending properties of spermidine supplementation remains vague, the activation of eukaryotic translation initiation factor 5A (eIF5A) via the posttranslational modification hypusination, plays an important role in the spermidine-mediated improvement of autophagy and mitochondrial function, building the basis of its geroprotective properties [7].

Despite the beneficial effects of spermidine supplementation on healthspan and longevity, polyamines have also been implicated in cancer cell growth [8]. ODC, the rate-limiting enzyme of polyamine anabolism, is transcriptionally activated by the oncogene *MYC*, suggesting that cellular transformation involves an upregulation of polyamines. Indeed, polyamine metabolism is often dysregulated in cancer cells [9], which can amount to a polyamine “addiction” of certain cancers, such as pancreatic ductal adenocarcinoma [8]. Consequently, some therapeutic approaches targeting the transport, anabolism or catabolism of polyamines, most notably the specific inhibition of ODC with the inhibitor difluoromethylornithine (DFMO), have been explored to treat neoplastic diseases [8, 10].

The notion that spermidine and polyamines in general are essential for cancer cell proliferation has been regarded as a major caveat in dietary supplementation regimes. However, epidemiological studies challenge this paradigm as they indicate a negative correlation of dietary spermidine intake and cancer-related mortality [11, 12]. Despite decades of research, pharmacological inhibition of polyamine production did not lead to cancer therapies for patients so far [10, 13]. Conversely, growing body of evidence suggests that spermidine has important cell-autonomous and non-autonomous cancer-preventive functions, especially through enhanced anticancer immunosurveillance, i.e., the efficient removal of malignant cells by the immune system [14]. The Janus-faced role of spermidine in cancer vs. other cells warrants a closer look at the molecular targets and their respective role in normal and transformed cells and tissues.

## THE MOLECULAR TARGET SPACE OF SPERMIDINE

The molecular target space of spermidine can be categorized into three classes: (1) non-specific interactions; (2) site-specific, orthosteric and allosteric interactions; and (3) covalent binding (**Table 1**).

**Table 1.** Direct molecular targets of spermidine.

**Target**	**Mode of action**	**Affinity measure**	**Oncogenic role of target**	**Anti-cancer role of target**	**References**
eIF5A	Covalent modification (hypusination)	K_m_ of deoxyhypusine synthase, K_m_ = 6.49 µM	Essential for MYC and LSD1 translation in cancer cells	Promotes autophagy through TFEB translation and mitochondrial protein expression in immune cells	[55, 56, 75]
EP300	Competitive inhibition	n.d. (∼40% inhibition of H3^K56^ acetylation observed with 10 µM spermidine)	Suspected to promote aberrant activity of enhancer elements. Inhibition of autophagy	Involved in expression of tumor-suppressive circular RNAs	[43, 46, 47]
MTP	Allosteric activation	K_d_ of HADHA = 0.10 μM	ATP supply in tumors relying on oxidative energy metabolism	Promotes mitochondrial function in CD8^+^ T cells	[2, 50]
hCA	Inhibition	K_i_ = 0.11 µM	pH buffering in acidic tumor microenvironment	n.d.	[30, 32]
NMDAR	Allosteric activation	n.d.; ∼50% increase of noncompetitive antagonist binding with 30 µM spermidine	Stimulates proliferation through ERK1/2 signaling and p53/p21 downregulation	Important for T cell receptor signaling in CD4^+^ and CD8^+^ T cells	[36, 38, 39]

eIF5A = Eukaryotic translation initiation factor 5A-1, EP300 = E1A Binding Protein P300, hCA = human carbonic anhydrase, HADHA = hydratase subunit A, MTP = mitochondrial trifunctional protein, NMDAR = N-methyl-D-aspartate receptors. n.d. = not determined.

### Non-specific interactions of spermidine

Due to its positively charged amine groups, spermidine interacts in a counterion-like, non-selective fashion with negatively charged biomolecules, which might represent a primordial function of polyamines. Indeed, early studies found spermidine bound to phosphate-containing molecules such (e.g., DNA, RNA, phospholipids, ATP) [15] with dissociation constants (K_d_) between spermidine and DNA (K_d_=0.49 µM) similar to those of the typical DNA-associated ion Mg^2+^ (K_d_=0.65 µM) [16]. Although the biophysical basis for these interactions is well understood [17, 18], the biological consequences are not, possibly owing to the fact that it is difficult to pinpoint the effects of spermidine treatment to such, rather unspecific, molecular interactions. Still, there is increasing evidence that spermidine has a regulatory function through non-specific interactions, for instance, via the induction of structural changes in RNA or tRNA anticodon modification [19, 20]. One proposed role of spermidine is the protection of DNA against mutagens, e.g., singlet oxygen, especially during DNA replication [21]. In support of this hypothesis, yeast cells treated with spermidine are protected from different DNA-damaging noxa, like UV light and ethyl methanesulfonate [22]. In general, polyamines are effective reactive oxygen species (ROS) scavengers [23, 24], which protects healthy cells on the one hand, but may also impede ROS-mediated regulated cell death in cancer cells.

Sequence-specific interaction of spermidine with DNA can enhance binding efficiencies of transcription factors *in vitro*, e.g. nuclear factor κB (NF-κB) and estrogen receptor α (ERα), which are both implicated in breast cancer [25, 26]. Experiments with uranyl photo-probing demonstrated that polyamines preferentially interact with bent adenine stretches, which are also found close to NF-κB binding sites [27]. However, it is not understood whether these interactions are specifically required in cancer cells to enable proliferative transcription programs. Importantly, spermidine can also inhibit the interaction of transcription factors with DNA, at least *in vitro*, e.g., in the case of the malignant stem cell factor Oct-1 (encoded by the gene *POU2F1*) [25], and thereby potentially exerts tumor-suppressive activity in this specific setting [28].

### Site-specific interactions of spermidine

Importantly, the alkyl chains of spermidine (and other polyamines) also mediate structural interactions (e.g., hydrogen bonds and hydrophobic interactions) in addition to the ionic, electrostatic forces [17, 29]. Hence, spermidine binds other cell components, such as proteins, in a specific fashion (beyond the apparent interaction with enzymes in polyamine metabolism). For instance, spermidine inhibits human carbonic anhydrase (hCA; inhibitor constant, K_i_=0.11 µM) [30] through hydrogen bond interactions with the protein and the coordinated Zn(II) ligand [31]. Carbonic anhydrases, especially isoform IX, contribute to pH regulation and the resistance to cancer cell-borne local acidosis in the tumor microenvironment [32]. However, a therapeutic use of spermidine against carbonic anhydrases might be problematic in certain scenarios: in glioblastoma, for example, tumor-associated myeloid cells (but not infiltrating CD8^+^ T cells) upregulate polyamine synthesis to buffer low intracellular pH in the acidic microenvironment. DFMO feeding in tumor-engrafted mice perturbs the immunosuppressive tumor microenvironment and ultimately improves survival [33].

Spermidine is also an allosteric activator of membrane proteins, such as neuronal glutamate-activated N-methyl-D-aspartate receptors (NMDAR), critical for memory formation [34–36]. Inferred from experiments with spermine, spermidine likely binds to a subunit-subunit interface between the GluN1 and GluN2B subunits of the NMDAR and thereby stabilizes the dimer in an active conformation [37]. We have previously shown that dietary spermidine supplementation improves spatial memory in aged mice, which, however, does involve signatures of increased hippocampal NMDAR signaling [6]. Interestingly, NMDARs are expressed in different cancer cell types, even in cells of non-neuronal origin, and are suspected to activate the mTOR pathway though autocrine and paracrine signaling [38]. NMDAR signaling also plays a crucial role in T cells, e.g. in fine-tuning Th1 versus Th2 cell responses [39]. The consequences of spermidine treatment on cancer- vs. immune cell-associated NMDAR signaling have not been explored so far. Data from experiments with NMDAR agonists and antagonists, respectively, suggest a positive role of NMDAR activation on anticancer immunosurveillance but – at the same time – also on tumor progression: NMDAR antagonists elicit anticancer action through the inhibition of extracellular signal-regulated kinase 1/2 (ERK1/2) pathway and an upregulation of the tumor suppressor proteins p21 and p53 [38]. Vice versa, they also reduce T cell receptor signaling both in CD4^+^ and CD8^+^ T cells, resulting in decreased cytotoxic T lymphocyte action towards cancer cells [40]. NMDAR agonists shift Th cell populations towards Th2-like cells by promoting cell death predominantly in Th1-like cells [39].

The first findings on the anti-aging properties of spermidine implicated post-translational protein modifications in the beneficial effects of spermidine supplementation, particularly a reduction of protein lysine acetylation [41]. Indeed, spermidine treatment in colon carcinoma HCT116 cells decreases cellular protein acetylation [42], an effect attributed to the competitive inhibition of the protein acetyl-transferase EP300 [43]. In other settings, i.e., treatment of lymphocytic Jurkat cells, spermidine did not de-acetylate the EP300 targets histone 3 and autophagy-related protein 7 (ATG7) [4]. Intriguingly, somatic mutation data in bladder cancer indicates that nonsense and missense mutations of *EP300* are associated with signatures of improved antitumor immunity, and overall favorable clinical prognosis [44]. Early reports suggested a tumor-suppressive function of EP300 [45], and at least in some cancer types, e.g. colorectal cancer, reduced EP300 expression is implicated in the attenuated expression of tumor-suppressive circular RNAs [46]. Still, accumulating evidence points at a oncogenic role in many scenarios, for instance in acute myeloid leukemia [47] or esophageal squamous carcinoma [48]. In line with these data, genetic knockdown of EP300 reduces metastatic capacity in xenograft mouse models of triple negative breast cancer and chemical inhibition of histone acetyltransferase activity increases sensitivity to radiotherapy in head and neck squamous cell carcinoma harboring potentially gain-of-function type mutations in *EP300* [49].

In their recent paper, Al-Habsi *et al.* added another player to the spermidine target space: Mitochondrial trifunctional protein (MTP), an enzyme complex that mediates the enoyl-CoA hydratase, 3-hydroxyacyl-CoA dehydrogenase, and 3-ketoacyl-CoA thiolase activities of mitochondrial fatty acid oxidation (FAO) [2]. In detail, the authors demonstrate that spermidine allosterically activates MTP by binding to both HADHA and HADHB subunits, with a K_d_ of 0.10 μM (for HADHA). The low Michaelis-Menten constant (K_m_) of spermidine-mediated MTP activation (0.4-0.7 μM) compared to the substrate affinity of deoxyhypusine synthase (K_m_=6.49 µM) tempts the speculation that the activation of FAO might contribute to the acute effects of spermidine treatment. MTP activity is crucial for the energy demand of CD8^+^ T cells and accordingly, spermidine i.p. injections enhanced antitumor immunity [2]. Pharmacological MTP activation in the context of cancer should be carefully adjusted to the specific tumor bioenergetics, especially the reliance on anaerobic vs. oxidative energy metabolism. For instance, oxidative lung tumors can be treated with the MTP inhibitor trimetazidine, as they rely on FAO for ATP supply [50].

### Covalent binding of spermidine

Spermidine is the sole substrate for the hypusination of eIF5A, a unique post-translational modification, which activates the protein [51]. Hypusinated eIF5A facilitates the translation of polyproline (and other sterically challenging) motifs and is required to express mitochondrial proteins efficiently [52]. Interfering with eIF5A hypusination severely impacts immune cells by reducing oxidative phosphorylation-dependent alternative activation of macrophages [52] and CD4^+^ helper T cell polarization [53]. Interestingly, aging-dependent B cell senescence has been linked to reduced spermidine levels and concomitant hypusination insufficiency [4]. In B cells, hypusination is required for transcription factor EB (TFEB) expression, which promotes lysosomal function and autophagy [4].

Beside the crucial role in immune cell function, eIF5A hypusination promotes neoplastic growth at multiple levels (reviewed in [54]), including several positive feedback loops: Multiplex gene expression analysis in colorectal cancer cells showed a feedback mechanism of hypusination on the expression of *MYC* (which indirectly augments hypusination by promoting polyamine biosynthesis). Hypusinated eIF5A resolves ribosomal stalling at distinct pausing motifs in the *MYC* coding sequence, and thereby promotes tumor growth [55]. The transcriptional coactivator Yes-associated protein (YAP) and transcriptional coactivator with PDZ-binding motif (TAZ) are additional factors that promote ODC expression. Hypusinated eIF5A supports the transcriptional repression of tumor suppressors by YAP/TAZ by enhancing the translation of lysine-specific histone demethylase 1 (LSD1), which demethylates histone H3 [56].

Translation control by spermidine (and other polyamines) is also vital for polyamine auto-regulation. Messenger RNAs of enzymes and regulators of polyamine biosynthesis have distinct sequence features that allow fine-tuning of protein synthesis based on polyamine levels [57]. Mechanistically, high polyamine levels modulate ribosomal scanning and frameshifting, likely by interfering with eIF5A function [58]. The role of this interference with eIF5A (as opposed to the positive regulation by increased hypusination) in the context of spermidine supplementation has yet to be investigated.

## ANTI-CANCER EFFECTS OF SPERMIDINE VS. GROWTH-PROMOTING ACTIVITY

The tumor-promoting role of polyamine metabolism, which has been extensively reviewed elsewhere [10], demands a careful dissection of the risk profile of spermidine based on the available preclinical and clinical data: Lifelong supplementation of 3 mM spermidine to the drinking water did not lead to enhanced occurrence of tumors in male C57BL/6 mice [3], and i.p. injections of spermidine in mice with chemically provoked breast cancer did not show any difference in tumor mortality [59]. Furthermore, chemically induced liver cancer and fibrosis, as well as colon carcinogenesis, can be partially suppressed by simultaneous spermidine administration [60, 61]. Spermidine injections enhance the antitumor efficacy of chemotherapy in mice in an autophagy-dependent fashion [14]. These preclinical findings are supported by prospective and case-control studies, indicating lower tumor-related mortality [11] and colorectal cancer risk [12, 62] in individuals with higher dietary polyamine intake.

**Figure 1:**
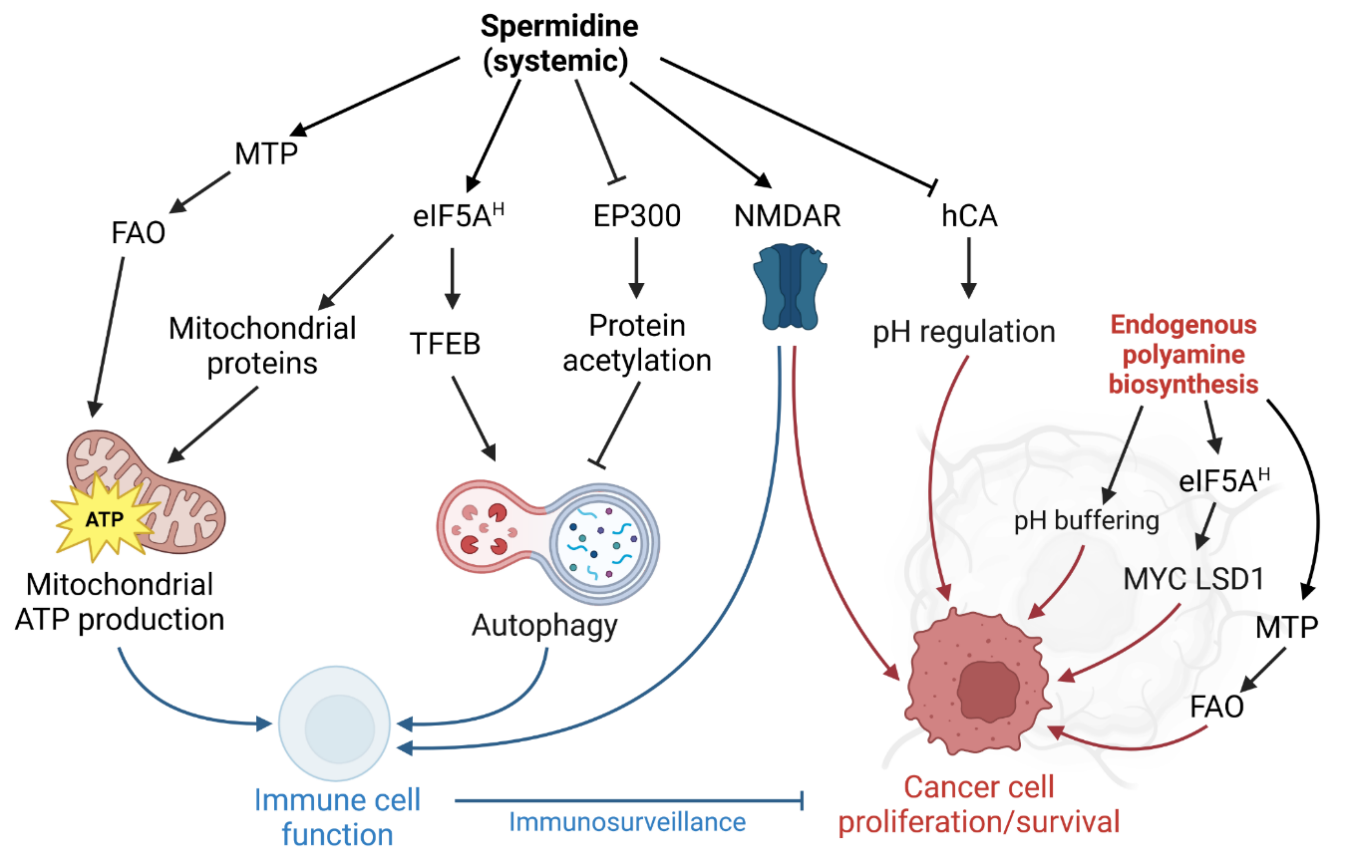
FIGURE 1: Model for spermidine-mediated effects in immune and cancer cells. Systemically supplied spermidine elicits antitumor responses predominantly through targeting pathways that improve immune cell function (blue arrows). However, in cancer cells or the tumor microenvironment regulation of these targets by endogenous polyamine biosynthesis may also promote proliferation and cancer cell survival (red arrows). See text for details. MTP, mitochondrial trifunctional protein. FAO, fatty acid oxidation. eIF5A^H^, hypusinated eukaryotic initiation factor 5A. TFEB, transcription factor EB. EP300, histone acetyltransferase E1A binding protein P300. NMDAR, N-methyl aspartate receptor. hCA, human carbonic anhydrase. LSD1, lysine-specific histone demethylase 1. ATP, adenosine triphosphate. Illustration generated with Biorender.

One possible explanation of the discrepancy between the anti-tumorigenic and tumor growth-promoting functions of spermidine might be the role of cell-autonomous vs. non-autonomous polyamine supply. We speculate that the anti-tumorigenic activity of spermidine is predominantly mediated *in trans* by improved immune responses. We thus speculate that intracellular spermidine production might promote proliferation, whereas external administration might activate the immune response in a fashion that overrides the tumor-promoting function (**Figure 1**). An important factor might be the administration route: While tumors might acutely benefit from enhanced polyamine biosynthesis *in situ* and elevated localized polyamine concentrations, a systemic administration might favor immune cell responses. Indeed, oral administration via the drinking water is sufficient to modulate polyamine-dependent pathways in B and T cells in mice [4, 63]. Nevertheless, since clinical trials of spermidine supplementation in the context of tumor growth are lacking, spermidine supplementation, regardless of the administration route, is not advised for cancer patients at this point. Future research should address which tumor-types (with a special focus on tumor bioenergetics) and which genetic predisposition or microbiome of the host might modulate anti-tumorigenic or potential tumor growth-promoting functions of spermidine supplementation [64]. Also, unconventional approaches such as combining localized DFMO treatment with systemic spermidine supplementation should be explored.

## ACUTE AND CHRONIC EFFECTS OF SPERMIDINE SUPPLEMENTATION

The pleiotropic target space of spermidine and the cell type-specific response to changes in polyamine metabolism pose questions about the acute and chronic effects of spermidine supplementation. As discussed above, MTP activation and the subsequent boost of FAO is a very early event in response to increased spermidine supply. Pathological scenarios involving disturbed lipid homeostasis might benefit from polyamine-targeted interventions. Previously, spermidine treatment was shown to ameliorate high-fat-diet-induced obesity via increased lipolysis in murine fat tissue [5]. Interestingly, these effects persisted in autophagy-incompetent mice, stimulating the speculation that MTP could be involved in these observations.

Interestingly, aged mice with heterozygous MTP deficiency develop hepatic steatosis, hyperinsulinemia and damaged hepatic mitochondria [65], signs of non-alcoholic fatty liver disease (NAFLD), one of the main risk factors for developing hepatocellular carcinoma [66]. In line, dysregulation of MTP is implicated in NAFLD pathogenesis in mice and humans [67, 68] and, consequently, FAO is impaired in such scenarios [66]. This bidirectional dependence is pharmacologically targetable and relevant, as – for instance – methionine restriction increases FAO, along with augmented levels of MTP-subunits HADHA/HADHB, which elicits hepatoprotective effects in *ob/ob* mice [69].

At the same time, increased hypusination and likely EP300 inhibition, for which no K_i_ values are available, might be more relevant for the chronic effects of spermidine supplementation. Indeed, one hour of spermidine treatment suffices to elevate FAO in naïve CD8^+^ T cells isolated from aged mice [2] and the effects of spermidine supplementation on eIF5A hypusination and its consequences have been primarily studied in longer-lasting experimental designs (days *in vitro* [4, 70], days to months *in vivo* [6, 71]). This is biologically plausible, given that several enzymes (DHPS, DOHH) need to transduce spermidine levels into activated/hypusinated eIF5A and subsequent translational events. Thus, to delineate the hierarchy of these molecular players, the cascade of effects on MTP, FAO, hypusination, and acetylation events should be studied *in vivo* and selected cellular models in a timely resolved excursion spanning a few hours to multiple days or weeks (in case of *in vivo* supplementation).

A particularly interesting question is how the acute and chronic effects of spermidine interact. For instance, the molecular eIF5A axis was found impaired in mice and humans suffering from non-alcoholic steatohepatitis (NASH), another risk factor for liver cancer, in a recent publication [72]. The same authors found that spermidine feeding ameliorated liver damage in a diet-induced NASH mouse model by normalizing FAO and mitochondrial function [72]. While MTP was not investigated in this publication, spermidine improved FAO in an eIF5A-dependent manner. Previously, it was shown several times that the spermidine-hypusination axis critically regulates mitochondrial function [6, 52, 71] and immune cell development [73, 74]. These findings raise interesting questions about the relationship between MTP and eIF5A. In a theoretical scenario, spermidine could acutely improve FAO via its interaction with MTP and maintain mitochondrial fatty acid utilization via eIF5A. However, this temporal hierarchy of events should be studied in coherent cellular and *in vivo* models.

## CONCLUSION

In terms of chemical properties, spermidine is not a complex molecule, which has fostered a widespread belief in its functional simplicity [20]. However, it becomes increasingly evident that spermidine exhibits highly specific molecular interactions with multiple targets (**Table 1**), with dedicated downstream cellular and physiological effects. In the quest for molecular effectors of spermidine, we may abandon the idea that single factors mediate the various beneficial effects described in pre-clinical literature. Moreover, the relative contribution of spermidine-responsive pathways might differ depending on the cell type or tissue of interest (as well as on crucial aspects of experimental timing). This is particularly apparent in immune vs. cancer cells, where spermidine either improves anti-tumor immunity or promotes transcriptional and translational processes to facilitate proliferation, respectively.

Future studies should evaluate the relative effects of different targets (e.g., by employing specific inhibitors), and consider divergent response kinetics depending on the target, as well as cell type-specific effects. Here, a standard panel of functional readouts, at least for NMDAR activity (when appropriate), translation efficiency, autophagy, mitochondrial respiratory function, FAO, and hypusination, could help to harmonize experimental setups and improve our understanding of spermidine-mediated health benefits across experimental designs in cellular, pre-clinical and clinical studies.

## AUTHOR CONTRIBUTION

A.Z. and S.J.H. wrote the first draft. All authors conceptualized the minireview, proofread, edited, and contributed substantially to the final version.

## Abbreviatons:

DFMO: – difluoromethylornithine;
eIF5A: – eukaryotic translation initiation factor 5A;
FAO: – fatty acid oxidation;
MTP: – mitochondrial trifunctional protein;
NAFLD: – non-alcoholic fatty liver disease;
NASH: – non-alcoholic steatohepatitis;
NMDAR: – neuronal glutamate-activated N-methyl-D-aspartate re-ceptor;
ODC: – ornithine decarboxylase;
ROS: – reactive oxygen species;
TAZ: – transcriptional coactivator with PDZ-binding motif;
YAP: – Yes-associated protein.
